# A Literature Review of the Numerical Analysis of Abdominal Aortic Aneurysms Treated with Endovascular Stent Grafts

**DOI:** 10.1155/2012/820389

**Published:** 2012-09-06

**Authors:** David Roy, Claude Kauffmann, Sébastien Delorme, Sophie Lerouge, Guy Cloutier, Gilles Soulez

**Affiliations:** ^1^Laboratoire Central de Traitement des Images, Research Center, Hôpital Notre-Dame, Centre Hospitalier de l'Université de Montréal (CRCHUM), Montréal, QC, Canada H2L 4M1; ^2^Department of Physiology, Biomedical Science Institute and Université de Montréal, Montréal, QC, Canada H3T 1J4; ^3^Industrial Materials Institute, National Research Council of Canada, Boucherville, QC, Canada J4B 6Y4; ^4^Department of Mechanical Engineering, École de Technologie Supérieure, Montréal, QC, Canada H3C 1K3; ^5^Department of Radiology, Radio-Oncology and Nuclear Medicine, Université de Montréal, Montréal, QC, Canada H2L 4M1; ^6^Département de Radiologie, Hôpital Notre-Dame, CRCHUM, 1560 Sherbrooke est, Montréal, QC, Canada H2L 4M1

## Abstract

The purpose of this paper is to present the basic principles and relevant advances in the computational modeling of abdominal aortic aneurysms and endovascular aneurysm repair, providing the community with up-to-date state of the art in terms of numerical analysis and biomechanics. Frameworks describing the mechanical behavior of the aortic wall already exist. However, intraluminal thrombus nonhomogeneous structure and porosity still need to be well characterized. Also, although the morphology and mechanical properties of calcifications have been investigated, their effects on wall stresses remain controversial. Computational fluid dynamics usually assumes a rigid artery wall, whereas fluid-structure interaction accounts for artery compliance but is still challenging since arteries and blood have similar densities. We discuss alternatives to fluid-structure interaction based on dynamic medical images that address patient-specific hemodynamics and geometries. We describe initial stresses, elastic boundary conditions, and statistical strength for rupture risk assessment. Special emphasis is accorded to workflow development, from the conversion of medical images into finite element models, to the simulation of catheter-aorta interactions and stent-graft deployment. Our purpose is also to elaborate the key ingredients leading to virtual stenting and endovascular repair planning that could improve the procedure and stent-grafts.

## 1. Introduction

Abdominal aortic aneurysm (AAA) rupture was the 14th leading cause of death in the USA in 2008 among white Americans aged between 60 and 85 years [1]. Still today, clinicians rely on 2 basic criteria before recommending surgery, that is, maximal diameter of 55 mm and growth rate over 5 mm every 6 months [2]. Patients with significant comorbidities are oriented toward “less invasive” endovascular aneurysm repair (EVAR) procedure, as opposed to the “classic” open surgery. Potential complications, such as endoleaks, migration, and occlusions, have raised concerns about durability after EVAR. During the last 30 years, much effort has been invested in improving our understanding of AAA and stent-grafts (SGs) biomechanics to prevent AAA rupture and optimize SG designs. We review the recent evolution of AAA and SG biomechanics, as well as the related computational analysis which is a powerful tool for decision making, and postoperative followup. The benefits of (validated) computational analysis stem in its flexible, accurate, and noninvasive nature.  Table 1 presents the main references quoted in this paper.

## 2. Purpose of AAA Modeling

Up to now, the clinical assessment of AAA rupture risk still ignores biomechanical factors. In reality, AAA will rupture when local stresses in the aortic wall reach its mechanical strength. These local stresses and vessel properties are influenced by a number of factors, so that the complexity of AAA biomechanics largely overpasses *Laplace's* law, which is strictly valid only for “perfectly” cylindrical tubes. There is a pressing need to clearly understand vascular biomechanics and develop tools to better model and predict vessel behavior. Eventually, such research will help to predict not only aneurysmal growth and AAA rupture risk, but also mechanical and physiological interactions between blood vessels and vascular implants (SGs) after EVAR, including blood rheology (hemodynamics).

To do so, that is, to properly simulate the physical properties of blood vessels, vascular implants, and blood flow, it is necessary to introduce mechanical and biochemical engineering concepts to the medical field. Capturing and simulating the complexity of AAA evolution and repair must be based on sound physics. Basic concepts are introduced in the following sections.

## 3. General Concepts of Biomechanics ****Applicable to Blood Vessels and Blood ****Rheology

**Figure 1:**
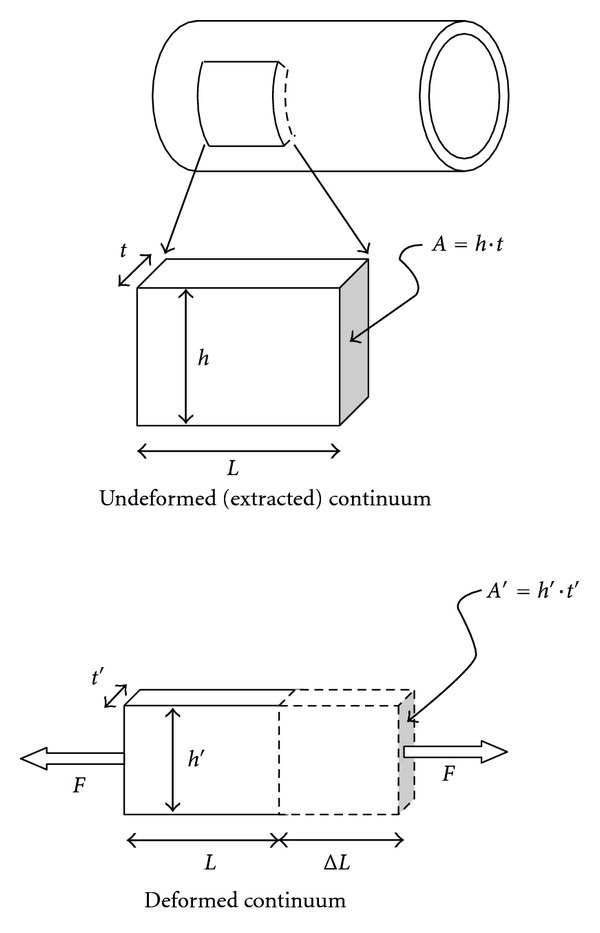
Stiffness definition.

We start with the basic definition of *stiffness* of any piece of material, namely a *continuum body*. When a spring is stretched, a simple equation describes force *F* required to extend it over a certain distance (Δ*L*):

(1) $$\begin{array}{c} F=k\Delta L, \end{array}$$

where *k* is *stiffness*. By analogy, this can be applied to a piece of material, such as a small blood vessel segment, as depicted in Figure 1. The following *normalized* (size-independent) equation prevails:

(2) $$\begin{array}{c} \frac{F}{A}=E\frac{\Delta L}{L}\;\text{or}\;\sigma =E\varepsilon , \end{array}$$

where *σ* = stress (“local pressure”) and *ε* = strain (“local stretch”). *E*, known as *Young's modulus,* can merely be interpreted as the *stiffness* of a* continuum body*. As can be seen, (2) is based on initial (undeformed) section area *A*. So-called *compliance*, or *softness*, is just the (exact mathematical) inverse of *stiffness*. Thus, the greater the *stiffness*, the lower the *compliance* and *vice versa*. 

**Figure 2:**
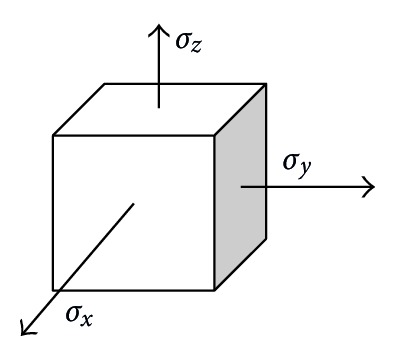
Multiaxial loading.

For material submitted to *multiaxial* loading, that is simultaneous tensile loads along 3 directions, as illustrated in Figure 2, *equivalent tensile* stress or *von Mises* stress (after Richard Edler von Mises) determines whether material strength is exceeded or not under given loading conditions. 

Actually, *von Mises* stress combines (into 1 single scalar value) not only individual tensile stresses but also shear stresses (also along 3 directions). Consider:

(3) $$\begin{array}{c} \sigma_{\text{VM}}=\sqrt{\frac{{\left(\sigma_{x}-\sigma_{y}\right)}^{2}+{\left(\sigma_{y}-\sigma_{z}\right)}^{2}+{\left(\sigma_{x}-\sigma_{z}\right)}^{2}}{2}}. \end{array}$$

**Figure 3:**
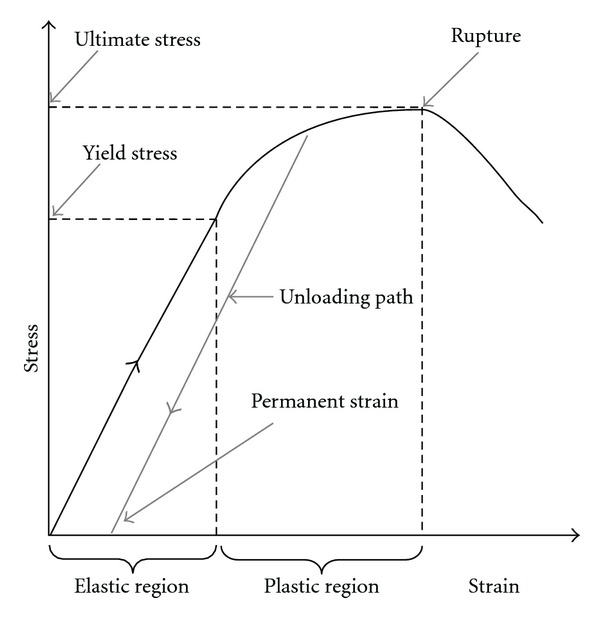
Yield and ultimate stresses.

As illustrated in Figure 3, a typical stress-strain curve (for “hard” materials) usually exhibits a (linear) “elastic” region delimited by *yield stress,* after which the *unloading path* leads to (*plastified*) permanent deformation/strain at zero stress, corresponding to irreversible damage. *Ultimate* stress is the maximum stress level supported after some *plastification* has occurred and corresponds to rupture. *Yield* and *ultimate* stresses define the strength of a given material. As per (2), *Young's modulus* is actually the slope of stress-strain curves. A given nonlinear material, such as a blood vessel, is thus not defined by a single *Young's modulus*, but rather by a modulus that depends on strain.

**Figure 4:**
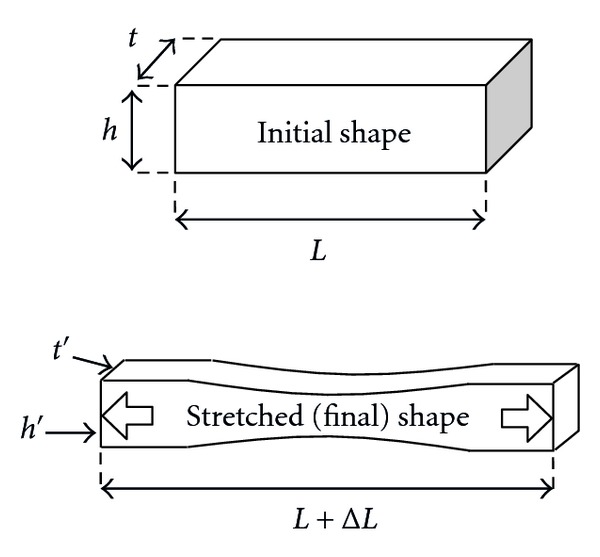
Poisson's effect.

*Poisson's ratio ν* is needed (along with *Young's modulus*) to completely define the mechanical behavior of simple materials. It is a *measure of transversal contraction* or necking of a stretched piece of material, as illustrated in Figure 4.

Poisson's effect can simply be observed when one stretches soft materials. Coming back to Figure 1, the exact definition of *Poisson's ratio ν* can now be given as the “ratio of transversal strain to strain along the stretched direction”:

(4) $$\begin{array}{c} \nu =\frac{\left(t-t^{\prime }\right)/t}{\Delta L/L}=\frac{\left(h-h^{\prime }\right)/h}{\Delta L/L}. \end{array}$$

Equations (1) to (4) are valid for both *tension* and *compression* load cases. 

It can be demonstrated that 0 ≤ *ν* ≤ 0.5, and most biological tissues, as well as rubber-like materials, exhibit *incompressibility* (i.e., the volume of deformed material remains constant), which is characterized by *ν* being close to (or equating) 0.5. In the case of biological tissues, *incompressibility* is understandable since they are mostly constituted of water, which is *incompressible* by nature. 

**Figure 5:**
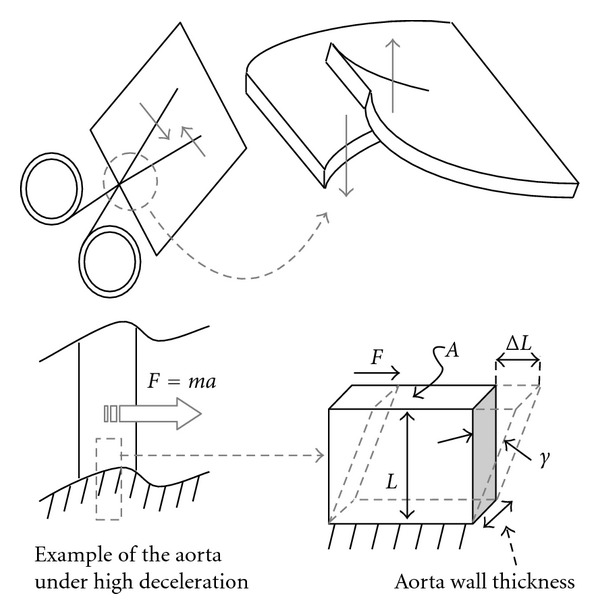
Shear stress.

*Young's modulus* and *Poisson's ratio* characterize the deformation of biological tissues and fibers undergoing *tension* or* compression* loads in all 3 directions, which is the “first mode of deformation”, typically caused by blood pressure in the case of blood vessels. The “second mode of deformation” is shear, typically caused by traumas or cuts, as depicted in Figure 5.

*Shear modulus G* is defined similarly to *Young's modulus*, with regard to the loading configuration presented in Figure 5. Shear stresses are forces acting “tangentially” per unit surface area (*A*):

(5) $$\begin{array}{c} \frac{F}{A}=G\frac{\Delta L}{L}, \end{array}$$

where *F*/*A* is shear stress, represented by *τ* in the literature, and Δ*L*/*L* actually corresponds to the deformation angle depicted in Figure 5, and represented by *γ* in the literature. In that case, Δ*L* is perpendicular to *L*, which is normal, because Δ*L* is considered as tangential displacement.

The *shear modulus* can be derived from *Young's modulus* and *Poisson's ratio* as follows:

(6) $$\begin{array}{c} G=\frac{E}{2\left(1+\nu \right)}\approx \frac{E}{3}\;\;\text{for biological tissues.} \end{array}$$

Shear stresses might make a significant contribution to AAA rupture due to AAA tortuosity, and especially when tangential forces are accounted for when simulating catheter introduction through iliac arteries. 

In the *International System *of Units, stresses, *Young's modulus* and *shear modulus,* are usually measured in *megapascal* (1 MPa = 1 N/mm^2^) or *gigapascal* (1 GPa = 1e03 MPa). In the *Imperial System,* the usual units are psi (pound per square inch), or ksi (1 ksi = 1e03 psi = 6.895 MPa).

There also exists *flow shear stresses *(FSS), which come from blood flow and exert “tangential” force on the inner face of blood vessels, as opposed to the earlier-mentioned shear stress that is more “structural”, that is, acting in the thickness of blood vessels. 

Although FSS are far lower than structural shear stresses, they play a key (physiological) role in the expression of enzymes on the sensitive *endothelium* cell layer. FSS participate in the vasoactive response of blood vessels, particularly in arterioles.

Mean flow shear force over a cardiac cycle and in the ideal case of a parabolic velocity profile is given by

(7) $$\begin{array}{c} F_{\text{mean shear}}=\frac{4\eta Q}{\pi R^{3}}, \end{array}$$

where *η* is dynamic viscosity (Pa*·*s), *Q* is the volumetric flow rate (mm^3^/s), *R* is mean blood vessel radius (mm).

Blood flow rheology can be classified as Newtonian or non-Newtonian. 

To illustrate the difference, water is a *Newtonian* fluid and paint is *non-Newtonian*, in the sense that stirring water with increasing intensity/velocity does not change its *viscosity*, whereas it does change for paint, which becomes more easily spreadable. Indeed, blood behaves similarly as paint, because of increasing *rouleaux* formation at low velocities (*i.e*., low shear rate conditions), as can be observed in the process of *coagulation*.

**Figure 6:**
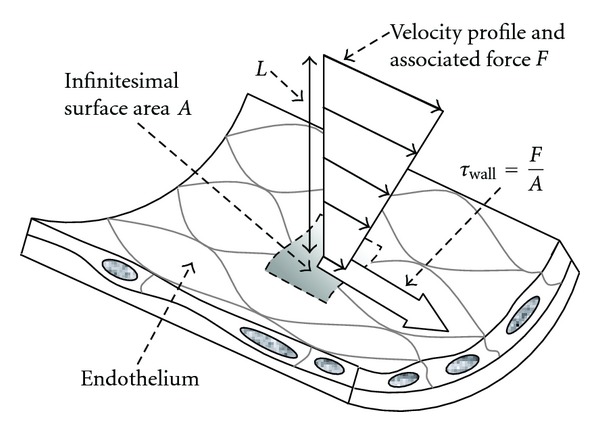
Flow shear stress.

For *Newtonian* fluids, shear stress is linearly related to blood shear rate via “constant” *viscosity*, whereas for *non-Newtonian* fluids, *viscosity* decreases as blood shear rate increases. This is illustrated in Figure 6 and by

(8) $$\begin{array}{c} \tau =\eta \left(\text{velocity}\right)\frac{\text{velocity}\left(L\right)}{L}, \end{array}$$

where *η*(velocity) is *dynamic viscosity*. 

Specialized literature [3] indicates that blood *viscosity* remains constant above a shear rate of 100 s^−1^, thus “*Newtonian*” approximation is valid only beyond this threshold, which also applies for pathological blood [4]. It is also indicated that the average velocity profile of blood in proximal arteries over a cardiac cycle is estimated to be 0.3 m/s with a maximum of 0.6 m/s. Considering mean shear rate as the velocity at the centerline of the artery to its radius (1 cm in average for a healthy aorta), the estimated mean shear rate is 0.6/0.01 = 60 s^−1^. The shear rate is minimum at the vessel center and maximum at the wall (equivalently the *non-Newtonian* property is emphasized at the center and minimized at the wall). Compared to the threshold of 100 s^−1^, it is clear that blood should (ideally) be considered as a *non-Newtonian* fluid, especially for AAA where re-circulations and possibly *disturbed* flows lead to low velocities and stagnation (which promotes erythrocyte aggregation), thus low shear rates and eventually high *viscosities*. For the sake of simplicity, the approximation of a *Newtonian* fluid is often made, but potential limitations should be borne in mind, especially when it comes to explaining the formation of *intraluminal thrombus* (ILT). Indeed, thrombus formation is wellknown to be promoted by low velocities and associated high *viscosities* [3]. Therefore, to explain their formation, it becomes important to consider a *non-Newtonian* formulation. 

The reader can also refer to a recent study that clearly showed the direct impact of increased erythrocyte aggregation on thrombus formation in femoral veins of experimented rabbits [5]. 

The relative importance of blood rheology was demonstrated after comparison between a *Newtonian *and *non-Newtonian* finite element models (FEMs) (based on *Carreau's* model) [6]. The difference in terms of FSS reached 42%, that is FSS = 1 Pa and 1.42 Pa for *non-Newtonian* and *Newtonian* models, respectively, both in AAA. As a matter of comparison, FSS of 1.95 Pa and 0.39 Pa were found in (healthy) *abdominal aortas* (AAs) and in AAAs, respectively, (with a *Newtonian* fluid assumption) [7].

FSS influence AAA growth in the long term [8]. FSS typically range from 1.00 to 16.69 Pa over a cardiac cycle for a *non-Newtonian* model, and from 0.51 to 16.11 Pa for a *Newtonian* model (in AAAs) [6], which does not mean that FSS distribution is the same between a *non-Newtonian* and *Newtonian* models for a given complex geometry. FSS are considered to describe the tangential forces exerted by blood (and fluids in general) on the inner face of vessels, while *von Mises* stresses denote the tridimensional stress state in vessel thickness.

**Figure 7:**
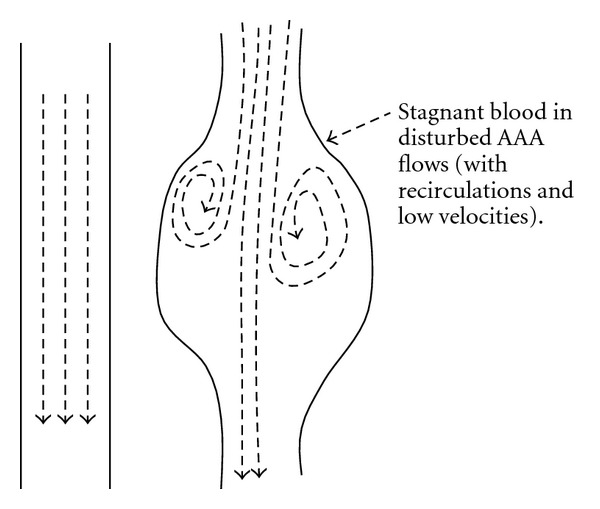
Laminar and disturbed flows.

In fact, it is known that FSS in *laminar* flow (see the healthy artery in Figure 7) stimulate the endothelial expression of a specific enzyme that produces the *nitric oxide*, which, in turn, acts simultaneously as vasodilator, prevents the aggregation of platelets (that tend to attract and stock lipid tissues), and, finally, generates anti-inflammatory substances [9].

## 4. Flow Modeling in Blood Vessels

Ideally, “full” *Navier-Stokes* equations (full description of mass, momentum, and energy conservation in fluid mechanics) should be solved to realistically characterize blood flow, accurately defining pressure and velocity profiles. Another challenge is considering fluid-structure interaction (FSI) (i.e., the mutual effect of flow and wall mechanics on each other). The key point is that more advanced formulations are needed, which is particularly true when modeling bifurcations such as the *circle of Willis*, the *carotid* artery, and AA, where pulsatile flow and wave reflection are to be accounted for. 

**Figure 8:**
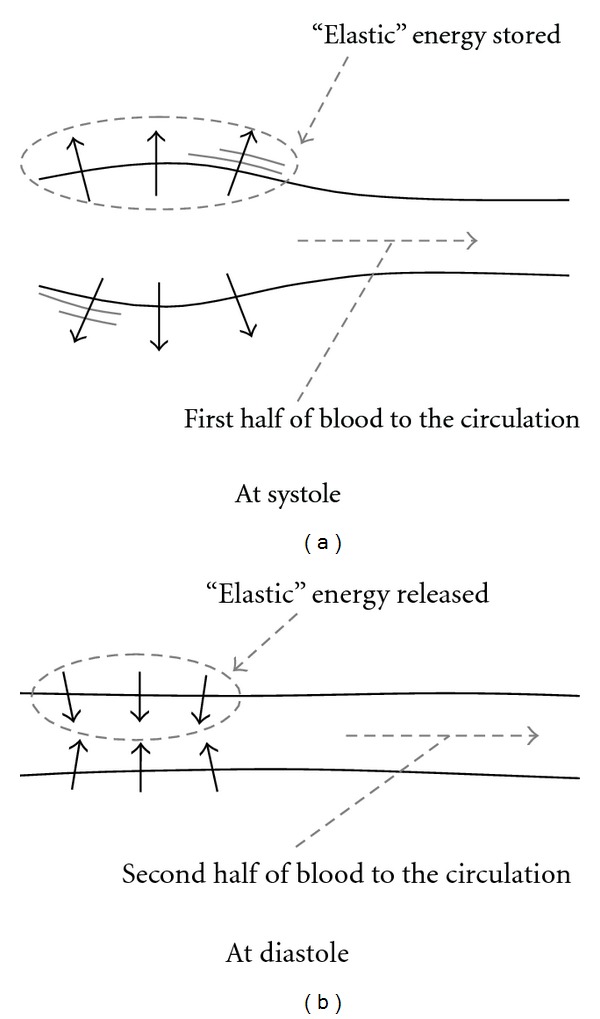
(a) Windkessel effect. (b) Cont'd Windkessel effect.

In reality, there is strong coupling between blood vessel deformation and blood flow. The *Windkessel* effect is a good illustration of this outcome (see Figure 8), where the *compliance* of heart-proximal arteries converts pulsatile flow into more constant/smooth flow. Indeed, after systole, one half of blood goes to the circulation and, thanks to its *compliance,* the artery stores “elastic” energy from the impulse, then the other half is forwarded during diastole when the artery releases its “elastic” energy. 

Another way to conceptualize arteries stiffness can be done via Peterson's elastic modulus (directly proportional to stiffness):

(9) $$\begin{array}{c} E_{p}=\frac{P_{\text{systolic}}-P_{\text{diastolic}}}{\left(D_{\text{systolic}}-D_{\text{diastolic}}\right)/D_{\text{diastolic}}}, \end{array}$$

where *P* and *D* stand for pressure and diameter, respectively. And again, the term stiffness is just the opposite concept of compliance (or distensibility).

Some authors [10] observed a significant decrease of AAA compliance after EVAR, along with AAA diameter reduction. Other investigations [11] showed increased impedance (vascular resistance to perfuse blood) in the case of grafted ascending and descending thoracic aortas, respectively, due to augmented forward wave and augmented reflections waves, as well as increased pulse pressure (difference between systolic and diastolic pressures). It was observed [11] that the more proximal the reduced compliance is located, the higher increase in pulse pressure, which is probably (also) due to altered *Windkessel* effect since the heart (left ventricle) has to compensate by an increased systolic pressure. 

Thus, the direct consequences of grafting and stent grafting are decreased compliance (equivalent to increased impedance), and systolic hypertension, all being proportional to heart proximity. 

Later in this paper, we will elaborate on FSI involving SGs.

## 5. Why We Need Finite Element Models

In the light of exposed biomechanical concepts, *Laplace's* law can be presented with a better understanding of its limitations. *Laplace's* law has been mathematically derived from a “perfectly” cylindrical shape; thus, it is valid only for pressurized tubes and cylindrical vessels. 

**Figure 9:**
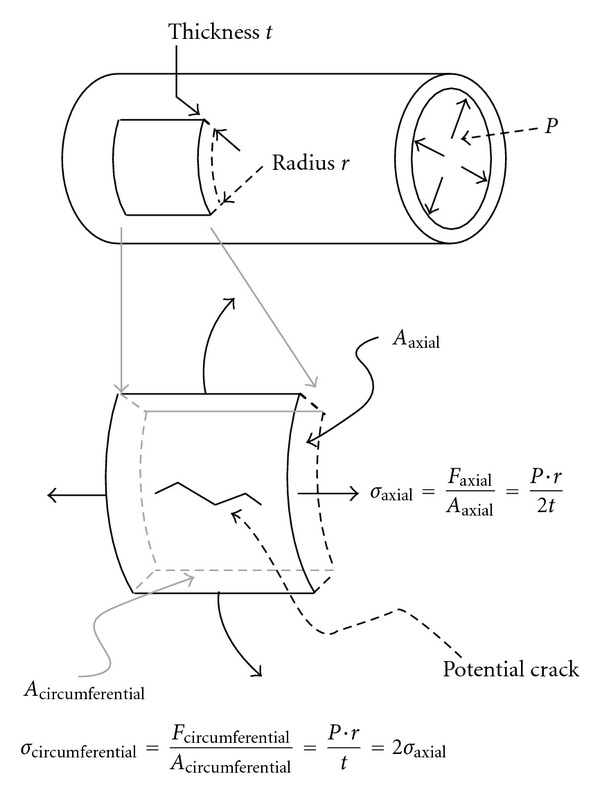
Laplace's law.

As can be seen in Figure 9, the predominant stresses predicted by *Laplace's* law are circumferentially oriented, and twice as large as axial stresses (explaining why cracks tend to appear axially). Circumferential stresses (or *hoop* stresses) are proportional to radius, and inversely proportional to thickness, which is why this formula is attractive to (“simplistically”) substantiate that AAAs are at higher risk of rupture when their radius increases (and thickness decreases). 

However, AAAs are tortuous and the real stress map is far more complex, as it is greatly sensitive to shape [12]. This can only be assessed by numerical simulations based on geometrical and mathematical *discretization* performed with *finite element analysis* (FEA). Alternatively, mechanical strain and shear modulus can be experimentally determined with novel ultrasound elastography methods, but validation still needs to be proven [13, 14].

In FEA, complex structures are *discretized* (cut) into small and then “simple-shaped” elements, such as small beams or shells for which analytical formulas and theories exist to predict their structural deformations and stresses. Eventually, elements and their individual contributions (deformations, stresses, temperature changes, etc.) are “continuously” assembled into a FEM *mesh*, providing “approximated” solutions to overall complex problems. Such solutions must *converge* as the elements are made even smaller (the FEM *mesh* is *refined*) to capture all geometric and structural (transitions between different materials) singularities. Typical singularities such as sharp angles, high gradients of thickness, or curvature might increase the stresses by a factor of 3 to 10, and the complexity of the required FEM.

The specific FEA concept of *boundary conditions *(BCs) merely refers to how a structure is attached and interacts with its environment, for example, the AA connects to the thoracic aorta and iliac arteries, remaining in contact with the spine and surrounding organs [15, 16].

There are 2 particular cases of FEA involving fluid flows, that is, computational fluid dynamics (CFD), which is the study of velocity and pressure evolution in flows (assuming a “perfectly” rigid conduit), and FSI, which is identical to CFD but goes further by accounting for the real *stiffness* of conduits. 

As a step prior to FEA [17, 18], “geometrical” *discretization/segmentation* is performed from medical image packages. The terms *discretization and segmentation* are common concepts in medical image analysis and FEA, as a way of representing a given geometry with quadrilateral or triangular plane (small) surfaces that create a so-called *mesh*. The difference is that a FEA *mesh* not only represents geometry, but also contains the physical formulation for structural analysis. 

There is no loss of accuracy when converting a *mesh* from any medical image package into FEA *mesh*, but quadrilateral surfaces (or *elements*) function more accurately and efficiently (than triangles for example). 

For further details, mathematically and physically inclined readers can refer to additional literature for solid mechanics [19, 20], FEA [21–23], and fluid mechanics [3, 24–27]. 

After these basic concepts, we detail *state-of-the-art* modeling of the aortic wall, as well as SG implantation. 

## 6. Specific Modeling of the Aortic Wall

### 6.1. Typical Stress-Strain Curves

**Figure 10:**
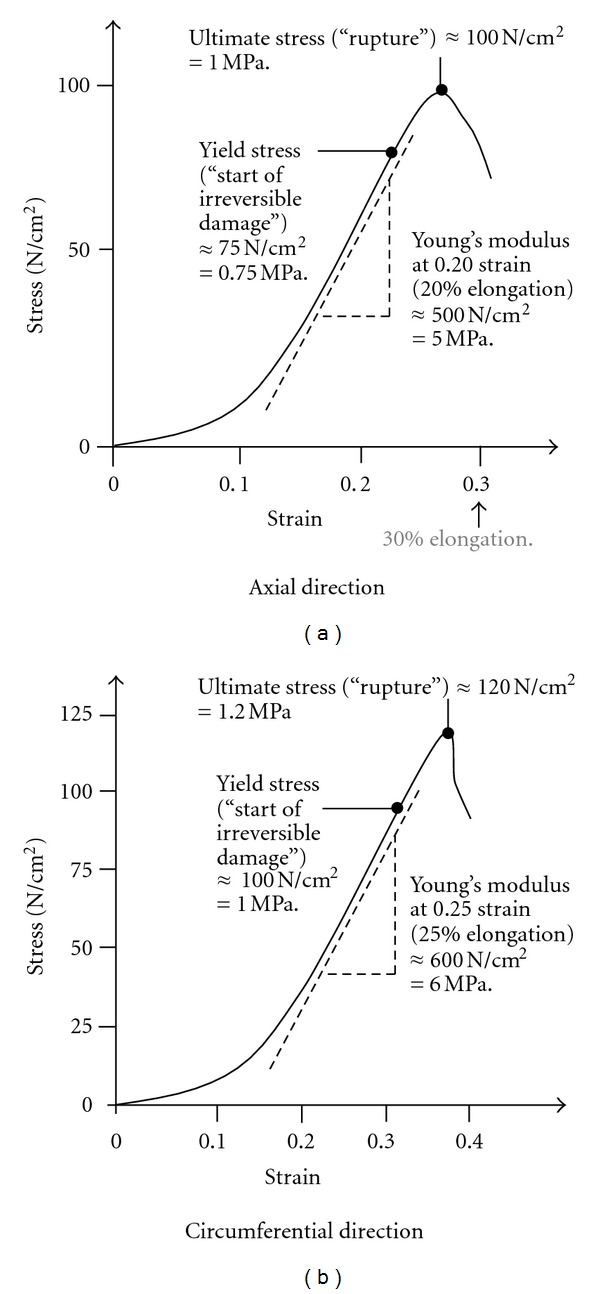
AAA stiffness [28].

One of the most comprehensive experimental determination of stress-strain curves of both AAAs and healthy aortas, in the axial and circumferential directions, goes back to 1996 [28]. *Yield* and *ultimate* stresses were identified for AAAs, that is 0.75 MPa and 1.00 MPa, respectively, in the axial direction, and 1.00 MPa and 1.20 MPa, respectively, in the circumferential direction (see Figure 10).

**Figure 11:**
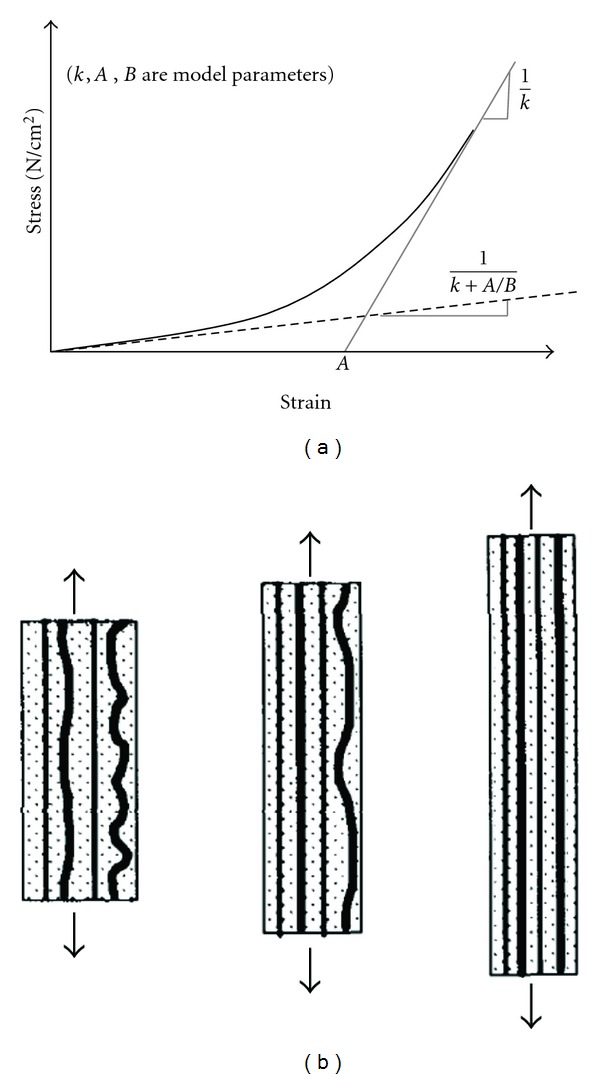
Recruitment of collagen fibers [28].

Based on these experimental data, alongside the definition of “recruitment” parameter “*A*” of collagen fibers (see Figure 11), which measures tortuosity, and also considering the contribution of total tissue *stiffness* by elastin and collagen, a first relatively simple mathematical model was defined. 

It was concluded that overall constitutive aorta tissue could be considered as *isotropic*, meaning that *stiffness* is the same in both axial and circumferential directions (as opposed to *anisotropic*) [28].

It should be pointed out that the stress-strain curves presented in Figure 10 are non-linear in the sense that the slope, and thus *stiffness*, is not constant, as opposed to “linear” materials that exhibit an almost constant slope (represented by a straight line and *Young's modulus* independent of strain). 

In 2000 another effort was done to define a dedicated framework that would also be suitable for FEA [29], which led to a 2-parameter, *hyperelastic* (non-linear elastic by essence), *isotropic*, and *incompressible* material model, based on a *strain energy density function *(SEDF). This model was validated with 69 freshly excised AAA samples. After undertaking sensitivity investigations FEA [29], it was also concluded that population mean values are accurate enough, even for clinical applications. In other words, there is no need to determine patient-specific mechanical properties (which is not yet affordable anyway) since the deviation from average values has no significant impact on numerical analyses. 

The same year, an intensive *histological* study stressed the “fiber-reinforced composite” structure of arteries, related to collagen fibers arranged helicoidally [30]. Then, on the basis of SEDF, a *hyperelastic*, *anisotropic*, and *incompressible* material constitutive model was defined and correlated experimentally. The importance of non-linear (collagen “recruitment”) and *anisotropic* (different *stiffness* in axial and circumferential directions due to collagen fiber orientations) models is now fully recognized. Later on, other anisotropic hyperelastic models were devised [31–33], all of them being correlated with experimental biaxial test data [34].

While *isotropic* models are appropriate for a first approximation [29], *anisotropic* models should be used for more accurate results [30]. Besides, the framework for the latter is already implemented in most FEA packages used in biomechanical research, making them easier to use and recommendable. 

When *anisotropic* materials are considered, local coordinates must be defined to account for AAA tortuosity, which ensures faithful material orientation [35, 36].

**Figure 12:**
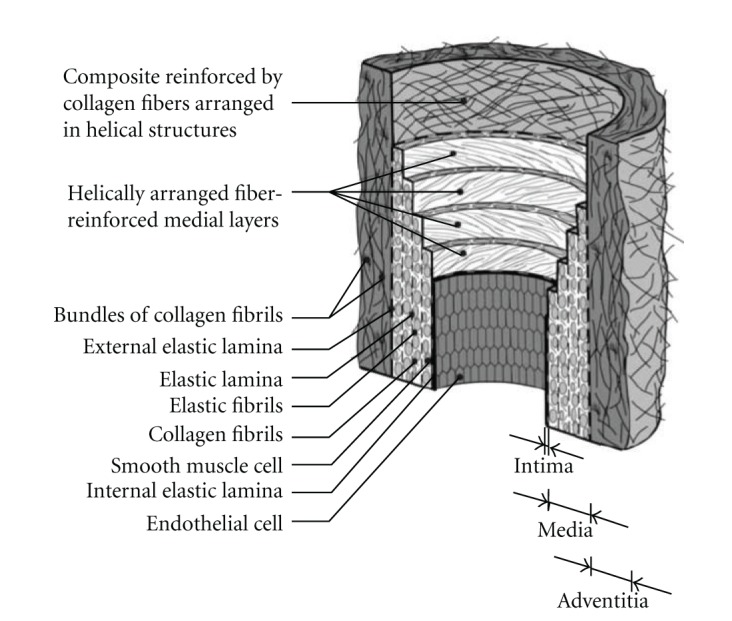
Arterial wall constitutive layers [30].

Here, it can be said that elastin (in the *media* layer) plays a key role in arterial *compliance* since it allows reversible, large, “elastic” deformations and makes vessels distensible. Consequently, decreased and even ruptured elastin in AAAs, as well as increased collagen, partly explains why they tend to extend irreversibly and become stiffer when (curly) collagen fibers (mainly in the *adventitia* layer) are taut. Eventually, the risk of rupture rises when growing stresses (due to increasing size and tortuosity) are not compensated any more by collagen increment.  Figure 12 depicts the typical structure of large arteries. 

### 6.2. How Wall Thickness Influences Stress Values

At this point, it must be underlined that to accurately predict wall stresses and, consequently, to properly assess rupture risk, better identification of the wall thickness map is still needed, as coarsely but fairly indicated by *Laplace's* law. This remains a major challenge for the *medical imaging *re-search community. The present consensus is to consider a thickness of 2 mm, but some authors [37] used 1 mm, which illustrates the degree of variability in current analyses.

### 6.3. Initial Stresses

Arteries are naturally pre-stressed (or “initially stressed”), which ensures their cylindrical shape, and they undergo additional stresses as they are loaded by blood pressure.

**Figure 13:**
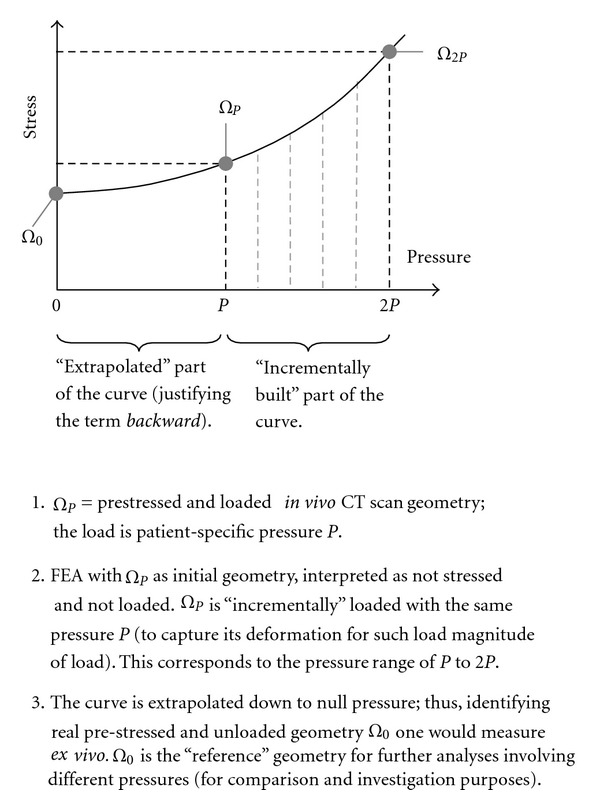
Backward incremental method.

Arterial geometries, extracted (*in vivo*) from *magnetic resonance imaging* (MRI) or *computed tomography* (CT) scans, are often considered as “unloaded” configurations on which blood pressure is applied directly. To correct this approximation and retrieve “true” unloaded geometry, the *backward incremental* (BI) method was introduced [38], as illustrated in Figure 13. BI is an iterative (numerical) method that starts from loaded arterial geometry, applying pressure perturbations until convergence occurs towards initial/unloaded geometry. The unloaded geometry was also called “zero-pressure” geometry [39]. The BI method is respectful of arteries non-linear behavior. 

“Zero-pressure” or unloaded geometries, however, present initial (or residual) “structural” stresses, which can be seen while arteries are being cut open. Thus, the terms “unloaded” and “unstressed” are not to be confounded. 

Neglecting to start any FEA from the real unloaded configuration leads to the overestimation of AAA deformation, overall stress level, and peak wall stress (around 20% error), as well as underestimation of FSS [17].

It is noteworthy that initial stresses were accounted for in the *anisotropic* model [30] of aortic wall referenced above (paragraph 6.1), but only in the circumferential direction, which is the dominant AAA growth direction.

### 6.4. Role of Calcifications

**Figure 14:**
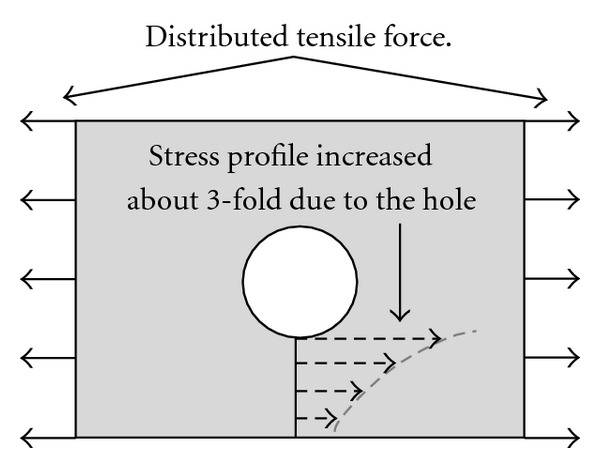
Stretched plate with hole.

Heterogeneity in wall structure, such as calcifications, can greatly influence stress values. Mechanical tests were conducted on calcified deposits from AAAs [40], motivated by the fact that calcifications were likely to act as local “stress concentrators” and increase rupture risk. Indeed, in structural stress analysis, any geometric singularity/irregularity leads to “stress concentration.” In engineering, holes in plates, notches, and sudden *stiffness* changes in materials act as stress concentrators, that is, increase stresses locally (see Figure 14 for illustration). There is an analogy in fluid mechanics where sharp angles, section-abrupt changes, and irregularities promote *disturbed* flow and shock wave propagation, exactly as with atherosclerotic plaques, AAAs, and bifurcations in abdominal and carotid arteries. 

Both the *micromorphology* and mechanical properties of vascular calcifications were analyzed [40], providing useful hardness and *Young's modulus* values. 

In 2010, a comparative investigation was conducted where AAAs had progressive degrees of calcification [41], that is, “noncalcified,” “disperse calcification,” “highly calcified,” and “pure calcification.” Corresponding stress-strain curves were charted. Pure calcifications were found to have *Young's modulus* superior to 40 MPa and a quasi-linear stress-strain behavior. Interestingly, it was observed that calcifications reduced average wall stress by up to 59.2%, as opposed to previous assumptions [42] that they would act as stress risers (up to 20% increase of stress). The authors doubted that calcified AAAs would heighten rupture risk, and do not believe that previously reported stress peaks are physiologically realistic as remodeling processes would probably attenuate them. 

### 6.5. Determining Rupture Risk: A Case-by-Case Study

Since stresses are highly sensitive to AAA (complex) geometry, distributed thickness, and material orientation (collagen fibers), there is no simple (analytical) formula to accurately assess stress levels, particularly when there are additional complexities such as calcifications. As in any other branch of mechanical engineering (aerospace, civil, etc.), every single geometrical irregularity such as thickness variation, multiple curvatures, openings, and tubular bifurcations can be considered as departures from perfect shapes, which cumulate their respective stress concentration factors, leading to high stress levels. Material heterogeneity also acts as stress raiser. In the case of tortuous AAAs, centerlines contribute to indicate the degree of irregularity [43], but are not sufficient *per se*. 

Therefore, “case-by-case” FEA studies are needed with accurate geometrical and material identification. A guideline is that calcifications should be accounted for when they are heavily present [38].

### 6.6. Rupture Risk Assessment

To properly ascertain the rupture risk, computed stresses must be compared to strength/allowable values, as is done in engineering with *margins of safety* (MS), which must remain positive to prevent rupture. Consider:

(10) $$\begin{array}{c} \text{MS}=\frac{\text{Peak\_Stress}}{\text{Allowable\_Stress}}-1. \end{array}$$

The key point here is that “local” *peak wall stress* (PWS) must remain inferior to “local” wall strength, to prevent rupture, simply because wall strength may vary significantly over the same AAA. For instance, having the PWS in an area where the local wall strength is superior does not indicate a rupture risk, but having a given wall stress (even less than PWS) in another area where the wall strength is smaller does indicate a real rupture risk. Therefore, relying only on the global PWS to identify locations of rupture is pointless [44]; what actually counts is the local ratio of stress over strength, knowing it is an added complexity to identify the wall strength map of a single AAA. 

Allowable stress values (usually* von Mises* stresses) must be defined, and the first obvious method is experimental, but cumbersome or even impossible if specific-patient values are needed for the sake of accuracy. Another trend is to infer global allowable values based on clinical statistics with relevant parameters such as AAA size, age, sex, smoking history [7, 45, 46]. However, such statistical AAA strength identification still needs large-scale data collection and correlation to be validated. 

It is worth mentioning that other authors [47–49] proposed a rupture criterion based on the average energy of interatomic bonds, but it is not trivial to appropriately define such average energy for the aortic wall.

## 7. Viscoelastic Properties of Blood Vessels

**Figure 15:**
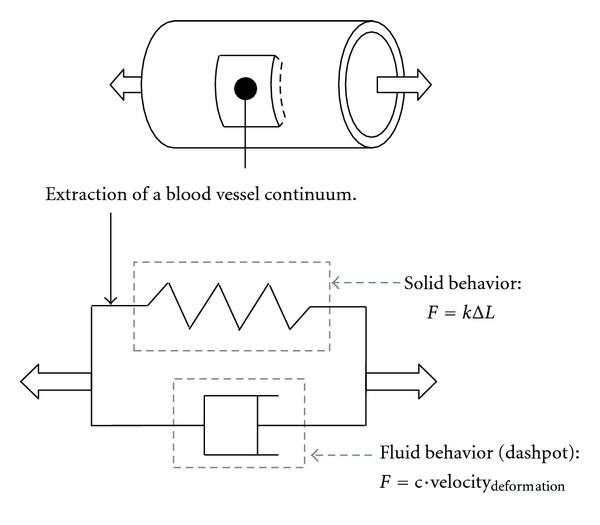
Viscoelasticity.

An additional complexity is related to the fact that biological materials exhibit more complex behavior than synthetic materials or metals. Indeed, the vessel wall exhibits non-linear elasticity and even *viscoelastic* properties, that is, properties that are time dependent. *Viscoelasticity* is a combination of solid and liquid mechanical behaviors (as some vehicle dampers and honey) and is represented in Figure 15 where *k* is “classic” *stiffness*, and *c* is the *damping coefficient* associated with the *velocity of deformation* (time dependent). The dashpot behaves like a leaky piston in a cylinder filled with a liquid of *viscosity η*.

Therefore, the structural response of *viscoelastic* materials is also time dependent. Since the “fluid” component relies on the *velocity of deformation*, it is relevant only when dynamic loads/events are to be analyzed. Otherwise *F* = *c* · velocity_deformation_ just reduces to 0. Not surprisingly, since biological tissues are mostly made of water, viscoelastic behavior may arise and become important under pulsatile contractility and relaxation motions. Typically, *ultrasound dynamic microelastography* can be used to characterize the viscoelastic properties of soft biological tissues [50].

Between 2003 and 2004, clarifications were made to the *viscoelasticity* of arteries, in relation to the proportion of *vascular smooth muscle cells* (VSMCs). Some authors [51] combined an *anisotropic* SEDF with a composite-like and *viscoelastic* formulation. But others [52, 53] indicated that *viscoelasticity* could be neglected for aortic modeling, since the aorta is a proximal artery with a large diameter and contains less VSMCs in the *media layer*, than medium-size vessels, such as femoral and cerebral arteries, which are considered *viscoelastic*. 

The assumption that aorta *viscoelasticity* can be neglected probably holds for static analyses, but should be accounted for in more detailed dynamic and FSI studies, because the *viscoelasticity* inherent to any biological tissue might modify the frequency response. 

## 8. Impact of Intraluminal Thrombus (ILT) on AAA Structural Growth, Pressurization, and Rupture Modeling

The ILT significantly contributes to the mechanical and biochemical modeling of AAAs. Therefore, it is worth including it in any comprehensive study.

The ILT was first, and still is, interpreted as a linear elastic material, with *Young's modulus* estimated to 0.11 MPa and *Poisson's ratio,* 0.45 [54–56]. However, in the early 2000s, detailed investigations of the ILT constitution revealed 3 layers along with their *Young's modulus*, that is, an (inner) *luminal* layer 0.54 MPa, a *medial* layer 0.28 MPa, and an *abluminal* layer, which was too degraded for any testing [57]. The *luminal* layer was stronger because it was made of freshly organized fibrin. These layers result from the organization of thrombus through aging. 

Simplified modeling of ILT as a linear elastic material led some authors to overestimate its “protective” effect and conclude that *von Mises* stresses in AAAs were reduced by up to 40%, whatever the material type (hyperelastic, viscoelastic, etc.) [58, 59]. In contrast, it was shown clinically [60] that pressure was almost constant throughout the ILT, which indicates an additional complexity since the actual porous nature of the ILT is to be accounted for.

In summary, the ILT, seen as a homogeneous and elastic material, indeed reduces AAA stresses by cohesion forces and by acting as a blood pressure “shield”, but its porous component mitigates the “cushion effect”, as a fraction of blood is actually transmitted to the AAA wall. Besides, the ILT reduces FSS (which generate anti-inflammatory substances), and may weaken the wall by *hypoxemia*. 

Therefore, a poro-elastic formulation might be more appropriate to realistically represent ILT mechanics, and such a model, based on Darcy's law, was presented in 2011 [61]. Though porosity and permeability values were published [62–64], a thorough clinical investigation is still needed to fully characterize ILT properties. 

Regarding the impact of ILT after EVAR, it was observed [65] that “sac shrinkage” decreases with ILT volume, which is not surprising since the ILT, depending on its compressibility, naturally offers a mechanical resistance to AAA remodeling. 

Furthermore, the ILT notoriously triggers hypoxia and inflammation at ILT/AAA interface, which constitutes a serious impairment to any AAA “elastic” reconstruction. 

Also, the ILT might still partially transmit the pulse pressure to the AAA wall and represent a type V endoleak (so-called endotension), but this occurs rarely, and some authors [66] showed that SG would mostly prevent such an event. 

Somehow, classic “open” surgery still presents an advantage in this aspect since the ILT is completely removed, and the AAA wall directly reshaped and stitched over the graft.

## 9. SG Modeling

Some studies might be oriented towards understanding how AAAs start and grow on their own, and others may focus on how the “SG + AAA + ILT” system interacts and remodels after EVAR, while undergoing blood pressure and flow. In the second type of study, proper modeling of SGs is important.

Most of the time, SG mechanical properties are reduced to linear and *isotropic* equivalent *Young's modulus* and *Poisson's ratio*. Here, the term “equivalent” refers to apparent/macroscopic properties, as opposed to detailed properties from the constitutive elements (stent and graft separately). Typical equivalent values are as follows: *E*_eq._ = 5.0 to 15 MPa and *v*_eq._ = 0.27, and *E*_eq._ = 50 MPa and *v*_eq._ = 0.45 (where the subscript _eq._ stands for “equivalent”) [67, 68]. 

There is a certain difficulty in simulating properly such devices, which are actually “composite” (metallic struts covered by polymeric grafts), presenting non-linear, *anisotropi*c, and nonuniform material properties. Therefore, such simplicity might become a serious limitation when SGs and their behavior (deformations) are to be studied in depth.

We believe that a more ideal model should faithfully represent the real *stiffness* map, even if doing so via a “patchwork” of equivalent properties.By “equivalent”, it must be understood that where the stent wire and graft are sutured; the 2 constitutive elements can be modeled as a simple stacking sequence, as is done with conventional composite materials. Another (“brute force”) alternative would be to completely represent SGs as they are, that is a combination of stent wire (modeled with beam elements) and graft (modeled with shell or membrane elements), but contact management between the stent wire and graft could become challenging. A model made with equivalent properties would present the following advantages:

- Universal model for different SG types/brands, without the need to rebuild FEM *mesh*, because equivalent materials are easy to update.
- Better efficiency because of less contact management (which is the third type of non-linearity with nonlinear materials and large displacements, resp.).
- Easier to integrate into FSI analyses, particularly in terms of contact, because an equivalent model is only produced with shell elements, rather than shell elements plus beam elements and their “suture” connections in the case of real representation.
- Pretension in the stent wires is easier to model (stent wires expand radially once separated from the graft).
- Contact of overlapping main body bifurcation with leg extension can be modeled efficiently.

In 2007, some authors [69] undertook mechanical tests on most used SGs and inferred their radial *stiffness*. They provided tabulated results, ready for further numerical analyses.

However, because of large variations in mechanical properties found in the literature, and because essential characteristics are missing, there is a need for exhaustive mechanical testing to validate theoretical models, especially when dealing with “large displacements”. 

Once a faithful SG model is achieved, the loads and pressures it will undergo must be assessed accurately in order to predict post-EVAR SG migration, and possibly improve its design. 

Following this goal, some authors [70] studied the impact of type II endoleaks on the intra-aneurysm sac pressure (after FSI analysis), based on an idealized AAA geometry. Blood rheology was represented with Quemada's model; the isotropic hyperelastic constitutive law from Raghavan and Vorp [29] was adopted for AAA mechanical behavior, and the SG was merely modeled as an isotropic linear elastic material with *Young's modulus* and *Poisson's ratio* of 100 MPa and 0.35, respectively. Also, a circumferential prestress equivalent to an oversize of 10% was applied at the proximal neck, and the SG was fixed to AAA wall. Eventually, *von Mises* stresses were evaluated to 0.23 and 2 MPa in the AAA wall and SG, respectively, and the intraluminal and intra-aneurysm sac pressures were found to be 121.5 and 62.7 mmHg, respectively. Last but not least, the vertical force (2.2 N) exerted on the SG was determined with the aim to anticipate migration, but this topic will be discussed with more details and other references in Section 10.

## 10. SG and AAA Contact Modeling

In a nutshell, the FEM provides a wealth of solutions to simulate contacts (with or without friction), and attachments between SGs and the AAA wall.

Probably the more challenging task lies in a preliminary understanding of the mechanical behavior of such devices. Radial tension (due to SGs oversizing) can be reproduced realistically, especially in landing zones. Also, type of element and *mesh* density (number of elements and nodes per line, surface, or volume unit) are key aspects of the model, since the algorithms managing contact work well only if basic guidelines are respected. For instance, contact operates between a pair of surfaces, the so-called *master* and *slave*. It is well known that the *slave* surface must have a higher density *mesh* than the *master*. Thus, the right combination/recipe must include all these aspects. 

If local attachments, for example, barbs and hooks, must be modeled, this is possible through constraining node *degrees of freedom* (DOF). Several contact definitions are possible, that is, “surface-to-surface” or “node-to-surface” (when connecting beam to shell elements, which is applicable to SG connection). Also, “self”-contact can be defined, as is the case of graft in large deformations. Friction coefficients can be accounted for. Finally, “tied” contacts, ensuring that 2 deformable entities remain linked, are useful to simulate sutures between the graft and stent. 

These types of connection will make it possible for full SG-AAA interaction to be modeled. 

Once a more realistic model is built, all kinds of “sensitive studies” (influence of varying a parameter on the overall model) become affordable to improve SG design. For instance, landing zone length or position could be optimized, different materials could be modeled, or even new attachment systems tested. Eventually, this will allow us to predict EVAR complications, such as endoleaks, migration, and kinking, in given patients.

## 11. Advancements in FSI Analysis and AAA**** Remodeling after EVAR

Accurate assessment of FSS via FSI analysis could actually help to understand the impact of EVAR on AAA remodeling. 

Structurally speaking, FSI faithfully simulates complex interactions between soft tissues, SGs, and pulsatile blood flow, which leads to more realistic blood pressure and velocity analyses, as well as wall stresses and displacements. 

In 2005 a significant study [71] included both SG and AAA in FSI, along with *Quemada's non-Newtonian* fluid model. This study showed that AAA peak wall *von Mises* stresses were reduced by a factor 20 once the artery was excluded from blood flow. This model, based on idealized/smooth AAA geometry and SG with uniform isotropic properties, was accurate enough to demonstrate the disturbed flow (with recirculations), identify pressure and velocity profiles, and drag force on SG. Surprisingly, although no accounting for a real patient-specific (tortuous) geometry, this model—before SG insertion—provided a peak wall *von Mises* stress of 0.59 MPa in iliac bifurcation area, where other authors [7] recently (2010) found 0.51 MPa with more sophisticated models that included a tortuous patient-specific geometry, along with ILT and calcifications. Having said that, the latter authors [7] where able to indicate additional critical areas in terms of *von Mises* stresses, which justify employing real geometries.

**Figure 16:**
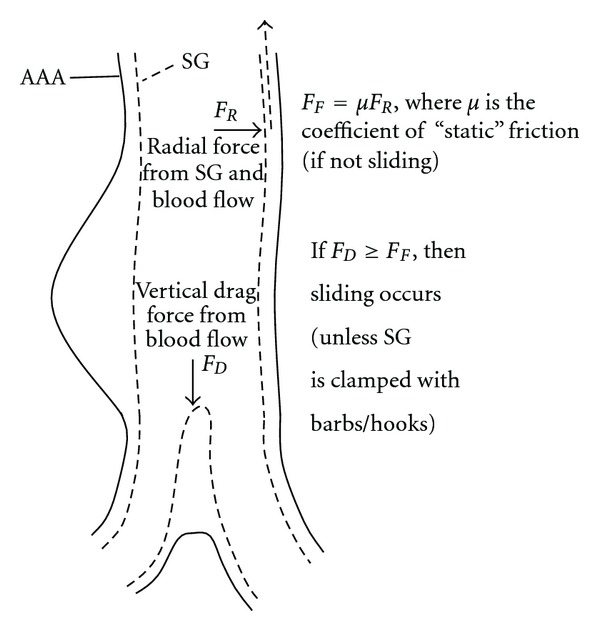
Definition of friction and drag forces.

In order to address SG migration due to blood flow, the coefficient of friction of SG was assessed experimentally [72]. Related forces of friction ranged between 3 and 12 N. However, since these tests were conducted without lubrication, the related forces of friction were probably overestimated, so one could expect forces of friction lower than 3 N. These forces of friction (or fixation forces for SGs) can be compared to drag forces computed after FSI analyses. A significant FSI study [73] was based on an idealized and quite tortuous AAA geometry (though variations of the proximal neck angle were also analyzed in this study), blood was interpreted as a *non-Newtonian* incompressible fluid (in a laminar flow) modeled with Quemada's rheological model, and the aneurysmal sac was filled with stagnant blood. AAA wall and SG were assigned isotropic linear material behaviors (with an equivalent *Young's modulus* for the SG) but some non-linear options were activated to account for large displacements. In terms of loads and boundary conditions, both a representative inlet velocity and outlet pressure profiles were imposed, and the SG was attached to the AAA wall at proximal and distal landing zones. Finally, a “prepressure” of 0.16 MPa was applied at the proximal neck contact between the AAA wall and SG to simulate the classic 15% of oversize. After FEM resolution, the drag force was computed from its tangential (linked to wall shear stresses) and normal components on the SG, with a value of 5 N. So, as depicted on Figure 16, comparing a drag force of 5 N with forces of friction of 3 N (or less with lubrication) clearly indicates a risk of migration and justifies designing SGs with hooks/bards. Knowing that proximal (and distal) neck angulations constitute a major risk factor of migration (indeed blood flow pushes on the SG not only tangentially but also normally), this type of FSI model is a very useful research tool that allows parametric geometries (neck angulations can be changed easily) to be analyzed, leading to valuable (and noninvasive) sensitivity results. 

Converging results were found after a study in which FSI analysis was performed with patient-specific AAA wall, including both an ILT and deployed SG [74]. The authors presented a peak wall *von Mises* stress of 0.38 MPa (before SG insertion), and a maximum drag force of 4.85 N on the SG.

FSI is also powerful to tackle endoleaks. We already mentioned a type II endoleak simulation via FSI in Section 9, but type I endoleaks, that can be caused by sharp neck angulations, nonoptimal oversizing, and insufficient neck length, were also investigated on highly idealized geometry in the proximal landing zone [68]. The authors included an ILT and performed FSI analysis with the *non-Newtonian* model from *Phan-Thien and Tanner*. Loss of contact at the proximal landing zone between the AAA wall and SG during the cardiac cycle could be predicted versus the following parameters:

- radial load exerted by the SG on AAA wall,
- proximal neck length,
- friction coefficient,
- AAA mechanical properties,
- ILT mechanical properties.

In particular, this study confirmed a minimum of 10% of oversize of the SG, as well as a similar stiffness between AAA and SG, to help avoiding type I endoleaks. 

Successful post-EVAR treatments are mostly assessed by aneurysm sac isolation from systemic pressure, and stabilization or reduction of AAA largest diameter. But how the modified AAA structure and hemodynamics influence AAA sac shrinkage remains incompletely understood [65]. Furthermore, how endoleaks might interfere with post-EVAR remodeling still needs to be fully addressed. However, recently, some authors [75] partially unveiled involved mechanisms, showing that not only aneurysm sac pressure was important, but also its pressure change over a period of time. Interestingly, Kwon et al. suggested that the intra-aneurysm sac pressure of 60 mmHg could be regarded as a threshold where AAAs remain stable and expand or shrink when their sac pressure, respectively, stands above or below this critical value. 

FSI computation remains challenging and time consuming. That's why a non-invasive method was devised to determine inlet velocity and outlets blood pressure over a cardiac cycle for given patients, along with corresponding wall geometry at different stages of cardiac cycle [76].This method was based on *dynamic magnetic resonance imaging *(MRI), and may be foreseen as an “easier” and direct alternative to FSI. In fact, a series of CDF runs were performed with variable geometric and physiologic data. Other researchers [77] considered the same technique to identify patient's aorta *compliance* and *distensibility*, by measuring pressure and volume changes. However, *ferromagnetic artifacts* induced by the stent struts were a major limitation. 

## 12. Virtual Stenting

The evaluation of soft tissue deformation and stress level during EVAR interventions is of great importance to clinicians, to predict appropriate deployment of SGs and optimize stent planning. The devices must be implanted accurately to avoid any complications, such as occlusion of renal arteries or limb thrombosis. This is an achievable goal since similar simulations [36] were successfully completed for other vascular regions such as coronary bifurcations.

## 13. Catheter Simulation

As a preliminary (and separate) step to virtual stenting, which actually would help predict SG positioning in AAAs after deployment, we believe in conducting analysis that would determine the “deforming” effect of catheter introduction into the artery. That way, virtual stenting simulation would “start” with updated geometry, realistically influenced/deformed by the catheter/intervention process. 

An overview of mechanical formulation and experiment of interaction between catheters and blood vessels can be found in literature [78]. Moreover, numerical simulation of catheter and artery interaction was successfully achieved via highly non-linear but manageable FEM reproducing the contact and progression of catheters along the iliac artery [79]. An *explicit solver* took 2 hours on a 4-CPU machine (64 bit) to analyze this FEM, which is a reasonable period of time.

Similarly, advanced FEM was created to simulate the deployment of a stent into a coronary bifurcation [36], showing the feasibility of such analyses, even if aortic SGs are far more complex.

## 14. Discussion

Since the first clinical implementation of EVAR in 1991 by Parodi, great advances have been made, particularly dedicated frameworks have been developed to characterize the mechanical behavior of arteries, and computational research has enhanced our understanding of arterial biomechanics and hemodynamics. Overall, this ongoing research has helped to improve both SG design and the implantation procedure. Nowadays, the trend is to include a greater number of mechanobiological aspects, such as more realistic ILT material, with porosity, initial stresses, calcifications, and realistic BCs accounting for surrounding organs. Recently, a “growth and remodeling” concept has been developed to capture elastin depletion and collagen production, leading to the concept of *fluid-solid-growth* numerical models.

However, every step is to be validated experimentally and clinically on a large scale. 

Although accounting for patient-specific geometry is now current, significant effort is still needed to accurately identify wall thickness from medical images, and incorporate patient-specific hemodynamics (pressure and velocity profiles). In addition, there is growing interest in simulating the interaction of catheters with AAA soft tissues to better predict perioperative SG positioning. 

Finally, “statistical strength” is a promising concept to assess rupture risk, but needs to be generalized and “cross-validated”. Probably, a deeper understanding of biomechanics is required to avoid relying only on statistics. 

## 15. Conclusion

EVAR is still an evolving area of research, and more efforts are needed to assist clinicians in taking decisions and orienting patients to surgery. 

Virtual stenting and the associated development of workflows will help interventional radiologists and surgeons plan EVAR interventions, and reduce the risks of complications. Moreover, such developments should help improve SG design. However, sufficient clinical validations will have to be conducted in parallel. 

The growing capability/memory of computers will allow several scenarios for given patients.

## Figures and Tables

**Table 1:** Relevant articles per category arranged chronologically.

	97	00	01	05	06	07	08	09	10	11	12
Materials (arteries, stent-grafts)		30, 68	29, 57		34, 73	55, 69	31	32, 33, 66	7, 72		
Conversion of segmented geometries into FEM					38		58	17	18		
Fluid-solid analyses with idealized geometries				71	68, 74						
Boundary conditions									15	16	
Initial stress					38						
Calcifications					40		58		7, 41		
Intra luminal thrombus	63	60	57				58, 59	56	62, 67	61	65
Endoleaks					70			68			
Fluid-solid analyses with stent-grafts								71	7, 74	75	
Patient-specific velocity and blood pressure profiles					76						
Statistical strength of arteries									7, 54		
Catheter simulation						78			79		
